# Safety, pharmacokinetics, and efficacy of belantamab mafodotin monotherapy in Japanese patients with relapsed or refractory multiple myeloma: DREAMM-11

**DOI:** 10.1007/s12185-023-03652-5

**Published:** 2023-09-05

**Authors:** Shinsuke Iida, Kazutaka Sunami, Yuko Mishima, Taku Fujii, Hitomi Kato, Takumi Terao, Yuki Matsuzawa, Mari Matsubara, Timothy Crossman, Brandon E. Kremer, Ira Gupta

**Affiliations:** 1 Department of Hematology and Oncology, Nagoya City University Institute of Medical and Pharmaceutical Sciences, Nagoya, Japan; 2 Department of Hematology, National Hospital Organization Okayama Medical Center, Okayama, Japan; 3 Department of Hematology Oncology, Cancer Institute Hospital, Japanese Foundation for Cancer Research, Tokyo, Japan; 4 GSK, Tokyo, Japan; 5 Oncology Global Clinical Development, GSK, Stevenage, UK; 6 Clinical Development, GSK, Upper Providence, PA USA; 7 Clinical Oncology, GSK, Upper Providence, PA USA

**Keywords:** B-cell maturation antigen, Belantamab mafodotin, Japan, Monotherapy, Relapsed/refractory multiple myeloma

## Abstract

Belantamab mafodotin, a B-cell maturation antigen-targeting antibody–drug conjugate (ADC), was investigated in Japanese patients with relapsed/refractory multiple myeloma in Part 1 of the phase I DREAMM-11 study. Patients who had received ≥ 2 prior lines of therapy including a proteasome inhibitor and immunomodulatory agent were eligible. Eight patients received belantamab mafodotin monotherapy at 2.5 mg/kg (n = 4) or 3.4 mg/kg (n = 4) by intravenous infusion every 3 weeks on day 1 of each cycle until disease progression or unacceptable toxicity. Primary objectives were tolerability and safety, and secondary objectives included pharmacokinetics (PK) and efficacy. The most common Grade ≥ 3 adverse event was thrombocytopenia/platelet count decreased (2.5 mg/kg cohort, 100% [4/4]; 3.4 mg/kg cohort, 75% [3/4]), and no dose-limiting toxicities were observed. Ocular events, including keratopathy findings, were observed in most patients (2.5 mg/kg cohort, 100% [4/4]; 3.4 mg/kg cohort, 75% [3/4]) and were managed with dose modifications. All resolved within the study period. Overall response rates were 50% (2/4) in the 2.5 mg/kg cohort and 25% (1/4) in the 3.4 mg/kg cohort. Although PK profiles in Japanese patients varied, individual exposures overlapped with previous results in Western populations. Belantamab mafodotin monotherapy was generally well-tolerated and demonstrated clinical activity at both doses.

**Supplementary Information:**

The online version contains supplementary material available at 10.1007/s12185-023-03652-5.

## Introduction

Multiple myeloma (MM) is a hematological malignancy involving the proliferation of terminally differentiated plasma cells [1]. The disease is characteristically accompanied by hypercalcemia, renal insufficiency, anemia, organ disease, and painful bone lesions [2], and patients with MM have poor health-related quality of life [1]. Despite substantial improvements in clinical outcomes since the introduction of proteasome inhibitors (PI), immunomodulatory agents, and anti-CD38 monoclonal antibodies (mAb), most patients with MM eventually relapse and/or become refractory to all available treatments [3, 4]. In addition, chimeric antigen receptor T-Cell (CAR-T) therapy targeting B-cell maturation antigen (BCMA) expressed on malignant plasma cells has shown clear evidence of efficacy in patients with MM [5] but high individual costs [6], an extended manufacturing process, and adverse effects (cytokine release syndrome and neurotoxicity) limit accessibility to CAR-T therapy in patients with MM [7].

Belantamab mafodotin is an antibody–drug conjugate (ADC) that targets BCMA, a cell-surface receptor expressed on multiple myeloma cells, but which is virtually absent on naïve and memory B-cells [8, 9]. The drug links the humanized IgG1 anti-BCMA mAb to a microtubule-disrupting agent, monomethyl auristatin F (MMAF or mafodotin) [10, 11], resulting in a multimodal mechanism of action. Mafodotin is delivered to BCMA-expressing MM cells, inducing apoptosis in association with the release of markers of immunogenic cell death [11]. The antibody component of belantamab mafodotin enhances immune effector cell-dependent mechanisms of action, such as antibody-dependent cellular cytotoxicity (ADCC) and phagocytosis (ADCP) [10].

The first-in-human DREAMM-1 study (NCT02064387) utilizing single agent belantamab mafodotin at 3.4 mg/kg administered intravenous (IV) every 3 weeks (Q3W) showed deep and durable responses in patients with RRMM [12]. Further evidence of clinical activity was demonstrated in the Phase II DREAMM-2 study (NCT03525678), at both 2.5 mg/kg and 3.4 mg/kg doses of belantamab mafodotin with a manageable safety profile [13]. At the 13-month follow up, an overall response was reported in 32% (31/97) of patients receiving 2.5 mg/kg while the estimated median duration of response, overall survival and progression free survival were 11 months, 13.7 months, and 2.8 months, respectively, in patients treated with 2.5 mg/kg Q3W of belantamab mafodotin [14]. At the data cut-off (January 2020), 10% (10/97) of patients receiving 2.5 mg/kg remained on study treatment. The DREAMM-2 study demonstrated a manageable safety profile in which the overall incidence of hematologic and infection-related adverse events (AEs) were low. The most common AE was keratopathy (including superficial punctate keratopathy) [14]. Such events are a known class effect of MMAF-containing ADCs [15–18]. Overall, belantamab mafodotin associated AEs were manageable with dose modifications/supportive care, with the 13-month follow-up data cut-off showing low rates of permanent discontinuation suggesting toxicity mitigation strategies can be successful [19].

Based on these findings, this Phase I study (DREAMM-11, NCT03828292) was conducted to evaluate the safety, efficacy, and pharmacokinetics (PK) of belantamab mafodotin monotherapy at two doses (2.5 and 3.4 mg/kg Q3W) in Japanese patients with RRMM previously treated with a PI and immunomodulatory agents as currently recommended by the Japanese Society of Hematology [20]. In addition, PK measures were included given the need to assess the PK profile in this population as interethnic differences in drug absorption, metabolism, and excretion may exist [21, 22]. This manuscript describes the dose escalation results (Part 1) from DREAMM-11 with a data cut-off date of November 2021.

## Materials and methods

### Patients

Eligible patients were ≥ 20 years of age, had histologically or cytologically confirmed MM [2], had an Eastern Cooperative Oncology Group (ECOG) performance status of 0–2; received ≥ 2 prior lines of therapy including PIs and immunomodulatory agents, were transplant ineligible or had received an autologous stem-cell transplant (SCT) more than 100 days prior to study enrollment, and had adequate organ system function. Patients with previous BCMA-targeted therapy, previous treatment with a mAb within 30 days, a history of allogeneic SCT and a current corneal epithelial disease (except for mild punctate keratopathy) were excluded (full inclusion and exclusion criteria in Tables S1 and S2).

### Study design

DREAMM-11 was a Phase I, 2-part (monotherapy [Part 1] and combination therapy [Part 2]), open-label, multicenter, dose escalation study investigating the safety, tolerability, PK, pharmacodynamics (PD), immunogenicity, and clinical activity of belantamab mafodotin in Japanese patients with RRMM. Patients received belantamab mafodotin 2.5 mg/kg or 3.4 mg/kg Q3W until disease progression, withdrawal of consent, or until unacceptable toxicity during the treatment period. Dose modifications were allowed for AEs, including thrombocytopenic events, and belantamab mafodotin associated ophthalmic exam or visual acuity findings if warranted. Administration of infusion-related reaction (IRR) prophylaxis prior to the first infusion of belantamab mafodotin was not mandatory but was permitted to be implemented according to clinical judgement. Preservative-free artificial tears were mandatory while corticosteroid eye drops were not required but could be used if clinically indicated per discretion of an ophthalmologist to mitigate ocular events.

### Endpoints and assessments

#### Safety and tolerability

The primary endpoints of Part 1 were to evaluate the safety and tolerability of belantamab mafodotin (number of patients with dose-limiting toxicities [DLTs], AEs, and changes in clinical signs and laboratory parameters). A DLT was defined as any observed toxicity in the first 21 days of the treatment cycle. DLT criteria considered related to belantamab mafodotin included the following: (1) Grade ≥ 3 febrile neutropenia lasting > 48 h; (2) Grade 4 thrombocytopenia with clinically significant bleeding; (3) any Grade ≥ 3 non-hematologic toxicity (other than ocular events) lasting > 48 h; (4) any Grade ≥ 3 non-hematologic laboratory value lasting > 48 h or with hospitalization; (5) Grade 4 ocular event per a protocol-defined scale which was specifically developed to combine ocular examination findings and visual acuity assessments into a single unified scale (Table S3); and (6) liver toxicity meeting protocol-defined stopping criteria. Subjects who were withdrawn from the study for reasons other than toxicity but prior to completion of the DLT observation period were replaced. AEs were graded by the investigator according to the National Cancer Institute (NCI)-Common Terminology Criteria for Adverse Events (CTCAE) (version 4.03). AEs of special interest (AESI) were ocular events, thrombocytopenia, and IRRs. Ocular events were reported by CTCAE and by findings following an eye examination administered by an ophthalmologist including visual acuity using a protocol-defined scale (Table S3). A safety and efficacy evaluation committee (SEEC) consisting of external doctors was formed in an expert advisory capacity to monitor patient safety. The SEEC could review study results, evaluate study conduct, and make recommendations to the Sponsor.

#### Efficacy

Clinical activity was included as a secondary endpoint of Part 1 and was measured by overall response rate (ORR, per International Myeloma Working Group [IMWG] response criteria, 2016 [23]) defined as the percentage of patients achieving confirmed partial response (PR) or better (≥ PR), and clinical benefit rate (CBR) defined as the percentage of patients with minimal response (MR) or better (≥ MR).

#### Pharmacokinetics and immunogenicity

Other secondary endpoints were the PK profiles of both belantamab mafodotin and cysteine-maleimidocaproyl monomethyl auristatin F (cys-mcMMAF); (area under the curve [AUC], maximum observed concentration [C_max_], time to C_max_ [t_max_], time to last measurable concentration [t_last_], clearance [CL], volume of distribution at steady state [V_ss_], terminal phase half-life [t_½_] [single dose], concentration at end of infusion [C_EOI_], trough plasma concentration [C_trough_], and accumulation ratio [R_ac_] [C_EOI_ and C_trough_] [repeat dose]) and anti-drug antibody (ADA) formation (incidence and titers) after IV single and repeat dose administration.

Blood samples for analysis of anti-belantamab mafodotin antibodies were collected at Day 1 of Cycle 1, 2, 3, 6, 9, 12, 16, and at end of treatment visit. Patients’ sera were tested for anti-belantamab mafodotin antibodies using a validated homogeneous acid dissociation bridging electrochemiluminescence immunoassay following a tiered-testing scheme consisting of screening, confirmation, and titration assays.

### Statistical analysis

The primary analyses (data cut-off 25 November 2021) were performed after both end of treatment (EOT) and completion of AE and serious AE (SAE) collection from the last patient. The sample size planned for this study arose from the pre-defined criteria for dose escalation and was not driven by statistical consideration. The maximum number of patients for Part 1 was 12: 6 patients each for the 2.5 mg/kg and 3.4 mg/kg cohorts based on the 3 + 3 design.

All eligible patients who received at least 1 dose of study treatment (‘All Treated’ population) were included in the safety, efficacy, and immunogenicity analyses. All patients fulfilling the ‘All Treated’ population criteria and having completed a minimum of 21 days or experienced a DLT before 21 days were included in DLT evaluation. Patients in the ‘All Treated’ population who had at least 1 non-missing PK assessment or 1 measurable PD sample analyzed were considered for PK/PD assessment.

Demographics, efficacy, and safety data were descriptively summarized. ORR and CBR for efficacy were reported with two-sided 95% exact confidence intervals (CI). Concentration–time PK data were plotted as linear and semi-logarithmic individual plasma concentration–time, mean, and median profiles; derived PK data were descriptively summarized.

Post-hoc analysis included calculating Revised International Staging System (R-ISS) disease stage from β_2_-microglobulin, serum albumin, high risk cytogenetics and lactate dehydrogenase level data collected.

## Results

### Patient disposition

Eight patients with RRMM were screened and administered belantamab mafodotin at either 2.5 mg/kg or 3.4 mg/kg dose Q3W (4 in each cohort). At data cut-off: 4 (100%) patients in the 2.5 mg/kg cohort and 3 (75%) patients in the 3.4 mg/kg cohort completed the study; 1 (25%) patient in the 3.4 mg/kg cohort died due to disease progression; no patients had withdrawn from the study; and all patients (100%) had discontinued the study treatment. Half (50%) of patients in the 2.5 mg/kg cohort discontinued due to progressive disease while the remaining patients discontinued due to AE and voluntary study withdrawal (1 [25%] patient each). All (100%) patients in the 3.4 mg/kg cohort discontinued due to progressive disease.

### Demographics and clinical characteristics

Patient demographics and baseline clinical characteristics are presented in Table 1. Median age was 65.5 years, 5 (63%) patients were male, and 5 (63%) of patients entered the study with ISS Stage I MM. The majority of patients had completed ≥ 5 lines of therapy (6 [75%] patients) and all were pretreated with regimens containing immunomodulators and PIs (8 [100%] patients for each) prior to being enrolled. In addition, 3 (38%) patients received an anti-CD38 mAb (daratumumab). Extramedullary disease was present in 3 (38%) patients and 3 (38%) patients had high risk cytogenetic features.

**Table 1:** Patient demographics and baseline clinical characteristics (All Treated population)

	2.5 mg/kg (n = 4)	3.4 mg/kg (n = 4)	Total (N = 8)
*Patient demographics*			
Sex, male, n (%)	3 (75)	2 (50)	5 (63)
Age (years), median (range)	63.0 (54–74)	68.0 (62–69)	65.5 (54–74)
*Baseline disease characteristics*			
ECOG performance status, n (%)			
0	2 (50)	2 (50)	4 (50)
1	2 (50)	1 (25)	3 (38)
2	0	1 (25)	1 (13)
ISS disease stage, n (%)			
I	3 (75)	2 (50)	5 (63)
II	1 (25)	0	1 (13)
III	0	2 (50)	2 (25)
R-ISS disease stage^a^, n (%)			
I	1 (25)	1 (25)	2 (25)
II	3 (75)	1 (25)	4 (50)
III	0	2 (50)	2 (25)
Prior lines of therapy, n (%)			
2‒4 lines	2 (50)	0	2 (25)
5 + lines	2 (50)	4 (100)	6 (75)
Type of MM, n (%)			
Nonsecretory	0	0	0
Secretory	4 (100)	4 (100)	8 (100)
Myeloma light chain, n (%)			
Kappa light chain	2 (50)	2 (50)	4 (50)
Lambda light chain	2 (50)	2 (50)	4 (50)
Myeloma immunoglobulin, n (%)			
IgA	0	0	0
IgD	0	0	0
IgE	0	0	0
IgG	2 (50)	4 (100)	6 (75)
IgM	0	0	0
Other^b^	2 (50)	0	2 (25)
Extramedullary disease, ^c^n (%)			
Yes	3 (75)	0	3 (38)
No	1 (25)	4 (100)	5 (63)
Lytic bone lesions, n (%)			
Yes	3 (75)	3 (75)	6 (75)
No	1 (25)	1 (25)	2 (25)
High cytogenetic risk type, ^d^n (%)			
High risk	0	3 (75)^e^	3 (38)
Other (non-high risk, not done, or missing)	4 (100)	1 (25)	5 (63)
ASCT, n (%)	3 (75)	3 (75)	6 (75)
Prior anti-cancer therapy, ^f^n (%)			
Steroids	4 (100)	4 (100)	8 (100)
Immunomodulator	4 (100)	4 (100)	8 (100)
Lenalidomide	4 (100)	4 (100)	8 (100)
Pomalidomide	0	3 (75)	3 (38)
Proteasome inhibitor	4 (100)	4 (100)	8 (100)
Bortezomib	4 (100)	4 (100)	8 (100)
Carfilzomib	3 (75)	3 (75)	6 (75)
Ixazomib	1 (25)	4 (100)	5 (63)
mAb	2 (50)	1 (25)	3 (38)
Daratumumab	2 (50)	1 (25)	3 (38)
Elotuzumab	1 (25)	0	1 (13)
HDAC inhibitor	0	2 (50)	2 (25)
Panobinostat lactate	0	2 (50)	2 (25)
Chemotherapy	4 (100)	3 (75)	7 (88)
Other^g^	0	3 (75)	3 (38)

ASCT, autologous stem-cell transplant; ECOG, Eastern Cooperative Oncology Group; HDAC, histone deacetylase; Ig, immunoglobulin; ISS, International Staging System; mAb, monoclonal antibody; MM, multiple myeloma; R-ISS, Revised International Staging System

^a^R-ISS was calculated from β_2_-microglobulin, serum albumin, high risk cytogenetics and LDH level data collected

^b^Including Bence Jones Protein (BJP)

^c^Including both bone-related and non-bone related EMD

^d^High cytogenetics risk type included any of genetic types of t(4;14), t(14;16), and 17p13del; the number of the genetic type was the number of patients with positive results only

^e^17p13del (*n* = 2) and 17p13del; t(4;14) (*n* = 1)

^f^Multiple categories per patient possible, total may add to more than 100%

^g^Including clarithromycin and investigational drugs

### Exposure

#### Treatment

Systemic exposure to belantamab mafodotin is summarized in Table 2. The median dose intensity per cycle was 2.355 mg/kg in the 2.5 mg/kg cohort and 2.823 mg/kg in the 3.4 mg/kg cohort. The median time on study treatment was 10.3 weeks in the 2.5 mg/kg cohort and 27.1 weeks in the 3.4 mg/kg cohort.

**Table 2:** Exposure to belantamab mafodotin (All Treated population)

	2.5 mg/kg (n = 4)	3.4 mg/kg (n = 4)	Total (N = 8)
Dose intensity per cycle (mg/kg)^a^			
Mean (SD)	2.308 (0.2345)	2.799 (0.5413)	2.553 (0.4668)
Median (range)	2.355 (2.02‒2.50)	2.823 (2.15‒3.40)	2.500 (2.02‒3.40)
Dose intensity (mg/kg/3 weeks)^b^			
Mean (SD)	1.740 (0.8303)	1.655 (1.3023)	1.697 (1.0121)
Median (range)	1.775 (0.94‒2.47)	1.283 (0.65‒3.40)	1.505 (0.65‒3.40)
Number of cycles, n (%)^c^			
Mean (SD)	6.3 (7.23)	4.5 (3.42)	5.4 (5.32)
Median (range)	3.0 (2‒17)	4.0 (1‒9)	3.5 (1‒17)
≤ 4 cycles	3 (75)	2 (50)	5 (63)
> 4 cycles	1 (25)	2 (50)	3 (38)
Duration of exposure (weeks)			
Mean (SD)	28.5 (41.24)	34.6 (35.71)	31.6 (35.86)
Median (range)	10.3 (3‒90)	27.1 (0‒84)	16.2 (0‒90)
Cumulative actual dose (mg/kg)^d^			
Mean (SD)	13.450 (14.1764)	11.440 (7.1716)	12.445 (10.4559)
Median (range)	7.500 (4.42‒4.38)	11.510 (3.40‒19.34)	8.910 (3.40‒34.38)

SD, standard deviation

^a^Cumulative actual dose/total number of cycles administered

^b^Cumulative actual dose/(last dose date-first dose date + 21)/21

^c^Cycles mean cycles dose given

^d^Cumulative actual dose (mg/kg) is the sum of dose at each cycle

#### Dose modifications

**Fig. 1:**
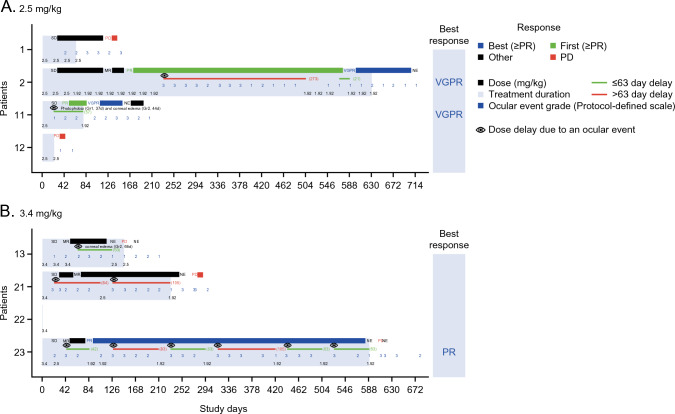
On-treatment duration and timing of response with PR or better. Patient response at each time point was assessed using IMWG 2016 criteria every 3 weeks regardless of dose delay. Dose delays due to any ocular event are indicated by the eye icon; ocular events per CTCAE (v4.03) are written out (highest grade, time [days] since 1st dose). Belantamab mafodotin doses (3.4, 2.5, or 1.92 mg/kg if 2.5 mg/kg was not tolerated) are also indicated. Treatment duration counts time difference between first dosing date and dosing end date without accounting for dose interruptions. CR, complete response; CTCAE, Common Terminology Criteria for Adverse Events; Gr, grade; IMWG, International Myeloma Working Group; MR, minimal response; NE, not evaluable; PD, progressive disease; SD, stable disease; VGPR, very good partial response

Five (63%) patients in total had at least 1 dose reduction or dose delay. Reasons for dose reductions were ocular events (3 [75%] patients in the 3.4 mg/kg cohort) and thrombocytopenia/platelet count decreased (2 [50%] patients in the 2.5 mg/kg cohort and 1 [25%] patient in the 3.4 mg/kg cohort). Primary reasons for dose delays were ocular events (3 [75%] in each cohort) and thrombocytopenia/platelet count decreased (1 [25%] patient in the 2.5 mg/kg cohort only). All dose reductions and dose delays were due to AEs (Tables S4 and S5). Reduction timing and dosage, and delay timing for each patient can be found in Fig. 1.

### Safety

#### Dose-limiting toxicities

Four (100%) patients in the 2.5 mg/kg cohort and 3 (75%) patients in the 3.4 mg/kg cohort were evaluated for DLTs; one patient in the 3.4 mg/kg cohort was excluded from DLT evaluation as they died (due to progressive disease) before completing the DLT observation period. No DLTs were reported in either dose cohort; 3.4 mg/kg was initiated after confirming the tolerability of 2.5 mg/kg.

#### Adverse events

All patients (100%) experienced at least 1 AE related to study treatment; 4 (100%) patients in the 2.5 mg/kg cohort and 3 (75%) patients in the 3.4 mg/kg cohort experienced at least 1 Grade 3 or 4 AE related to study treatment as judged by investigators; one patient in the 3.4 mg/kg experienced Grade 2 AEs only.

The most commonly reported (≥ 30% in either cohort) Grade ≥ 3 AE was thrombocytopenia (4 [100%] patients in the 2.5 mg/kg cohort and 3 [75%] in the 3.4 mg/kg cohort) (Table 3). Grade 4 AEs were reported in 4 patients in total: lymphopenia in 1 (25%) patient in the 2.5 mg/kg cohort; thrombocytopenia (2 [50%] patients), and lymphocyte count decreased (1 [25%]) in the 3.4 mg/kg cohort. No Grade 5 or fatal AEs were reported during the study. Two patients in the 2.5 mg/kg cohort experienced non-fatal SAEs during the study: one with Grade 2 pyrexia and the other with Grade 3 intratumoral hematoma which was resolving as of the last follow-up. Both SAEs were considered related to the study treatment by the investigator. No SAEs were reported in the 3.4 mg/kg cohort.

**Table 3:** Adverse events of all grades by preferred term (All Treated population)

Preferred term, n (%)	2.5 mg/kg (n = 4)		3.4 mg/kg (n = 4)		Total (N = 8)	
	Any grade	Grade ≥ 3	Any grade	Grade ≥ 3	Any grade	Grade ≥ 3
Any event	4 (100)	4 (100)	4 (100)	3 (75)	8 (100)	7 (88)
Thrombocytopenia^a^	4 (100)	4 (100)	3 (75)	3 (75)	7 (88)	7 (88)
Gamma-glutamyltransferase increased	1 (25)	1 (25)	1 (25)	1 (25)	2 (25)	2 (25)
Lymphocyte count decreased	1 (25)	1 (25)	1 (25)	1 (25)	2 (25)	2 (25)
Lymphopenia	1 (25)	1 (25)	1 (25)	1 (25)	2 (25)	2 (25)
Neutropenia	1 (25)	1 (25)	1 (25)	1 (25)	2 (25)	2 (25)
Hyperkalemia	0	0	1 (25)	1 (25)	1 (13)	1 (13)
Hyponatremia	0	0	1 (25)	1 (25)	1 (13)	1 (13)
Hypophosphatemia	0	0	1 (25)	1 (25)	1 (13)	1 (13)
Pharyngitis	0	0	1 (25)	1 (25)	1 (13)	1 (13)
Aspartate aminotransferase increased	2 (50)	1 (25)	0	0	2 (25)	1 (13)
Leukopenia	1 (25)	1 (25)	0	0	1 (13)	1 (13)
Anemia	1 (25)	0	1 (25)	1 (25)	1 (13)	1 (13)
Hypertension	1 (25)	1 (25)	0	0	1 (13)	1 (13)
Intratumoral hematoma	1 (25)	1 (25)	0	0	1 (13)	1 (13)
Neutrophil count decreased	1 (25)	1 (25)	0	0	1 (13)	1 (13)
White blood cell decreased	1 (25)	1 (25)	0	0	1 (13)	1 (13)
Liver disorder	0	0	1 (25)	1 (25)	1 (13)	1 (13)
Pneumonia bacterial	0	0	1 (25)	1 (25)	1 (13)	1 (13)
Diarrhea	0	0	2 (50)	0	2 (25)	0
Enterocolitis	0	0	2 (50)	0	2 (25)	0
Acute sinusitis	0	0	1 (25)	0	1 (13)	0
Conjunctival hemorrhage	0	0	1 (25)	0	1 (13)	0
Corneal edema^b^	1 (25)	0	1 (25)	0	2 (25)	0
Decreased appetite	0	0	1 (25)	0	1 (13)	0
Hyperemia	0	0	1 (25)	0	1 (13)	0
Iron deficiency anemia	0	0	1 (25)	0	1 (13)	0
Transfusion reaction	0	0	1 (25)	0	1 (13)	0
Visual impairment	0	0	1 (25)	0	1 (13)	0
Alanine aminotransferase increased	2 (50)	0	0	0	2 (25)	0
Infusion related reaction^c^	2 (50)	0	1 (25)	0	3 (38)	0
Blood alkaline phosphatase increased	1 (25)	0	1 (25)	0	2 (25)	0
Bursitis	1 (25)	0	0	0	1 (13)	0
Cardiac failure	1 (25)	0	0	0	1 (13)	0
Dyspnea	1 (25)	0	0	0	1 (13)	0
Epistaxis	1 (25)	0	0	0	1 (13)	0
Fatigue	1 (25)	0	0	0	1 (13)	0
Nasopharyngitis	1 (25)	0	0	0	1 (13)	0
Nausea	1 (25)	0	1 (25)	0	2 (25)	0
Photophobia^b^	1 (25)	0	0	0	1 (13)	0
Polyarthritis	1 (25)	0	0	0	1 (13)	0
Pyrexia^c^	1 (25)	0	1 (25)	0	2 (25)	0
Blurred vision	1 (25)	0	0	0	1 (13)	0
Vomiting	1 (25)	0	1 (25)	0	2 (25)	0

AESI, adverse events of special interest; CTCAE, Common Terminology Criteria for Adverse Events; IRR, infusion-related reaction

Displayed in descending order of Grade ≥ 3 total incidence by preferred term

^a^Thrombocytopenia includes the preferred terms thrombocytopenia and platelet count decreased as AESI

^b^AESIs ocular events (CTCAE)

^c^AESIs of IRR

AESIs of belantamab mafodotin included ocular, thrombocytopenic, and IRR events (Tables 3 and S6). Overall, 4 (100%) patients in the 2.5 mg/kg cohort and 3 (75%) in the 3.4 mg/kg cohort experienced an ocular event and 6 (75%) patients in total experienced a Grade ≥ 3 ocular event. Ocular events by CTCAE included corneal edema in 1 (25%) patient in each cohort (Grade 2) and photophobia in 1 (25%) patient in the 2.5 mg/kg cohort (Grade 1). Grade 3–4 ocular events by the protocol-defined scale (Table 4) were experienced by 3 (75%) patients in each cohort and most patients (3 [75%] in the 2.5 mg/kg cohort and 2 [50%] in the 3.4 mg/kg cohort) had temporary reduction in visual acuity. IRR events were reported in 2 (50%) patients in each cohort and included an IRR and pyrexia (1 [25%] patient in each cohort). No SAEs or deaths were associated with a thrombocytopenic event.

**Table 4:** Characteristics of ocular events by protocol-defined scale (All Treated population)

	2.5 mg/kg (n = 4)	3.4 mg/kg (n = 4)	Total (N = 8)
Number of patients with event, n (%)	4 (100)	3 (75)	7 (88)
Event characteristics, n (%)^a^			
Study treatment related	4 (100)	3 (75)	7 (88)
Maximum grade, n (%)			
Grade 1–2	1 (25)	0	1 (13)
Grade 3–4	3 (75)	3 (75)	6 (75)
Action taken, n (%)^b^			
Study treatment withdrawn	0	0	0
Dose reduced	0	3 (75)	3 (38)
Dose not changed	0	0	0
Dose interrupted/delayed	3 (75)	3 (75)	6 (75)
Dose reduced or interrupted/delayed	3 (75)	3 (75)	6 (75)

^a^Patients could be included in more than 1 category

^b^Patients were counted once under each action that was taken

### Efficacy

The ORR was 50% (95% CI: 6.8, 93.2) in the 2.5 mg/kg cohort and 25% (95% CI: 0.6, 80.6) in the 3.4 mg/kg cohort (Table 5). The CBR was 50% (95% CI: 6.8, 93.2) in the 2.5 mg/kg cohort and 75% (95% CI: 19.4, 99.4) in the 3.4 mg/kg cohort. In addition, some responders had a long treatment duration (the longest within their respective cohort) with 631 days (2.5 mg/kg cohort) and 589 days (3.4 mg/kg cohort) (Fig. 1).

**Table 5:** Overall response rate and clinical benefit rate

n (%)	2.5 mg/kg (n = 4)	3.4 mg/kg (n = 4)	Total (N = 8)
Best response			
Stringent complete response (sCR)	0	0	0
Complete response (CR)	0	0	0
Very good partial response (VGPR)	2 (50)	0	2 (25)
Partial response (PR)	0	1 (25)	1 (13)
Minimal response (MR)	0	2 (50)	2 (25)
Stable disease (SD)	1 (25)	0	1 (13)
Progressive disease (PD)	1 (25)	0	1 (13)
Not evaluable (NE)	0	1 (25)	1 (13)
Overall response rate			
sCR + CR + VGPR + PR	2 (50)	1 (25)	3 (38)
95% CI	(6.8, 93.2)	(0.6, 80.6)	(8.5, 75.5)
Clinical benefit rate			
sCR + CR + VGPR + PR + MR	2 (50)	3 (75)	5 (63)
95% CI	(6.8, 93.2)	(19.4, 99.4)	(24.5, 91.5)

### Pharmacokinetic and immunogenicity results

**Fig. 2:**
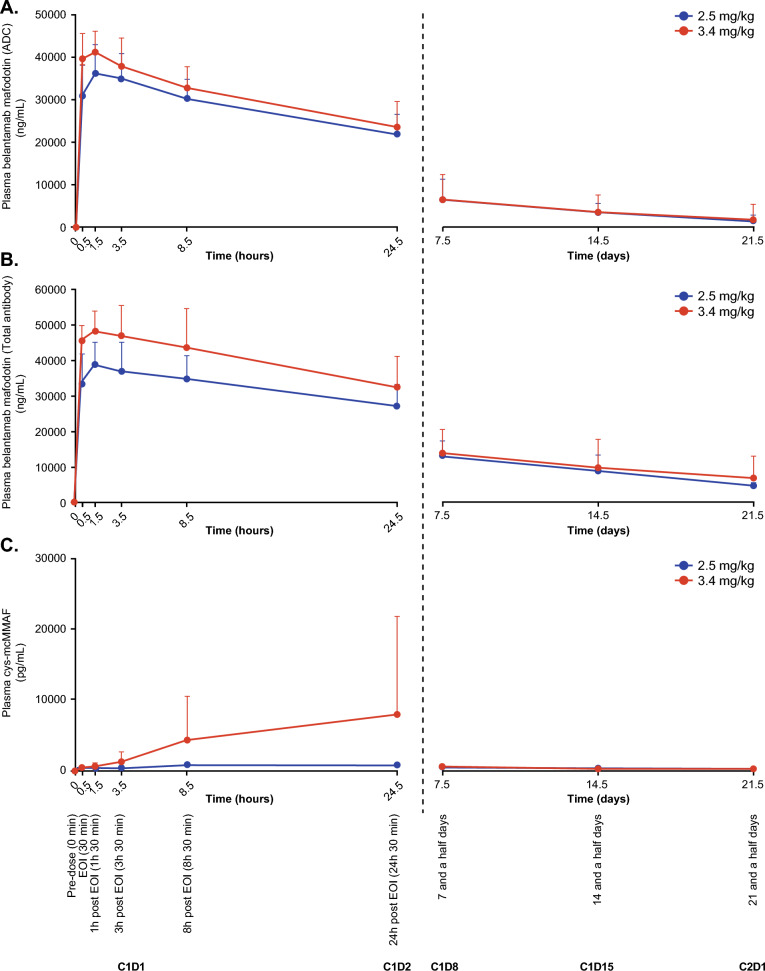
Belantamab mafodotin (ADC and total antibody) and cys-mcMMAF PK plasma concentration over time. Plasma concentrations of belantamab mafodotin ADC (A), total antibody (B), and cys-mcMMAF (C). Values are expressed as the mean + SD. Left panels report time in hours, right panels report time in days. ADC, antibody–drug conjugate; C, cycle; cys-mcMMAF, cysteine-maleimidocaproyl monomethyl auristatin F; D, day; EOI, end of infusion; SD, standard deviation

PK results are summarized for 3 analytes: belantamab mafodotin (ADC); total antibody (sum of ADC and naked mAb [without mcMMAF]) and cys-mcMMAF, (the microtubule inhibitor released from belantamab mafodotin during proteolysis [catabolism] of the mAb; Tables 6 and 7). Peak plasma concentrations of the ADC and total antibody were achieved by approximately 2.7 h in 2.5 mg/kg cohort and 1.6 h in 3.4 mg/kg cohort (Table 6), while peak plasma concentrations of the cys-mcMMAF were reached by approximately 16 h post dose for both cohorts (Table 7). t_1/2_ of the ADC and total antibody were comparable between cohorts. For the ADC, systemic CL was 35.0 mL/h and 45.3 mL/h and V_ss_ was 7.48 L and 8.22 L, in the 2.5 mg/kg and 3.4 mg/kg cohorts, respectively. Belantamab mafodotin (ADC and total antibody) and cyc-mcMMAF plasma concentrations over time for Cycle 1 are shown in Fig. 2.

All patients were negative for anti-belantamab mafodotin antibodies at baseline (N = 8) and post-baseline (N = 7).

**Table 6:** Summary of derived belantamab mafodotin (ADC and total antibody) PK parameters (Part 1, Cycle 1)

Parameter, geometric mean (% CVb)	Antibody drug conjugate (ADC)		Total antibody	
	2.5 mg/kg (n = 4)	3.4 mg/kg (n = 4)	2.5 mg/kg (n = 4)	3.4 mg/kg (n = 4)
C_-EOI_ (µg/mL)	30.3 (24.3)	39.4 (14.2)	32.7 (27.2)	45.7 (8.6)
C_max_ (µg/mL)	36.4 (19.1)	41.3 (12.8)	39.5 (17.0)	48.89 (14.1)
t_max_ (h)^a^	2.6 (1.6–3.7)	1.6 (0.7–1.7)	2.7 (1.6–8.5)	1.7 (0.6–3.7)
t_last_ (day)^a^	21.6 (15.1–22.1)	21.1 (1.0–22.0)	18.6 (14.1–22.1)	21.1 (1.0–22.0)
C_trough_ (µg/mL)	1.5 (29.4)^b^	1.4 (109.2)^b^	4.5 (22.2)^c^	5.2 (104.1)^b^
AUC _(0-tlast)_ (h*µg/mL)	3606.0 (22.6)	2392.3 (129.0)	5839.3 (33.0)	4046.1 (179.4)
AUC _(0-tau)_ (h*µg/mL)	3694.6 (26.3)	3807.9 (41.8)^b^	6358.4 (33.1)	7143.7 (45.6)^b^
AUC _(0-inf)_ (h*µg/mL)	4177.9 (28.5)	4243.2 (48.8)^b^	6939.1 (31.1)^b^	6247.4, 7378.0^c^
CL (mL/h)	35.0 (37.4)	45.3 (37.3)^b^	22.0 (34.8)^b^	21.7, 30.0^c^
V_ss_ (L)	7.5 (29.1)	8.2 (36.1)^b^	6.4 (18.8)^b^	4.6, 9.9^c^
t_1/2_ (day)	7.5 (9.0)	7.3 (27.6)^b^	9.0 (23.4)^b^	7.5, 11.6^c^

AUC, area under the curve; C_-EOI_, concentration at end of infusion; CL, clearance; C_max_; maximum observed concentration; C_trough_, trough plasma concentration; CVb, between-subject coefficient of variation; t_1/2_, terminal phase half-life; t_last_, time of last measurable concentration; t_max_, time to C_max_; V_SS_, volume of distribution

^a^Median (range)

^b^n = 3

^c^Individual values provided as n = 2

**Table 7:** Summary of derived cys-mcMMAF PK parameters (Part 1, Cycle 1)

Parameter, geometric mean (% CVb)	2.5 mg/kg (n = 4)	3.4 mg/kg (n = 4)
C_max_ (pg/mL)	748.9 (30.5)	2797.3 (326.5)
t_max_ (h)^a^	16.38 (8.55–24.58)	16.24 (8.45–24.1)
t_last_ (day)^a^	6.9 (6.8–14.9)	6.9 (1.0–7.0)
AUC _(0-tlast)_ (h*ng/mL)	73.3 (44.7)	141.9 (77.0)

AUC, area under the curve; C_max_; maximum observed concentration; t_last_, time of last measurable concentration; t_max_, time to C_max_; CVb, between-subject coefficient of variation

^a^Median (range)

## Discussion

This study was the first investigation in Japanese patients with RRMM to evaluate tolerability, safety, efficacy, and PK of belantamab mafodotin monotherapy. The doses selected for this study were 2.5 mg/kg and 3.4 mg/kg based on the previous studies, DREAMM-1 and DREAMM-2, which were conducted mainly in Western populations [12–14, 24]. Both the 2.5 mg/kg and 3.4 mg/kg doses were tolerated in Japanese patients. The safety profile was consistent with that previously reported in DREAMM-1 and DREAMM-2 for both dose cohorts [12–14, 24]. No new signals specific to the Japanese population were identified. Most common AEs included thrombocytopenic events and ocular events, consistent with DREAMM-1 and DREAMM-2 [12–14, 24]. AEs of Grade 3 and above were observed in almost all the patients (100% in the 2.5 mg/kg cohort; 75% in the 3.4 mg/kg cohort), which is comparable to DREAMM-2 (84% for 2.5 mg/kg) [14]. Only 1 AE led to permanent discontinuation of study treatment. One patient in the 2.5 mg/kg cohort discontinued treatment due to Grade 2 cardiac failure that occurred approximately 2 years after the first dose of study treatment which had not resolved at the last follow up. The investigator considered this AE to be related to the study treatment but was confounded by cardiovascular risk factors (hyperlipidemia and hypertension); however, cardiac failure was rarely reported in previous studies [12–14, 24]. AESI in this study included ocular events, thrombocytopenic events, and IRRs.

Ocular events have been observed in patients treated with belantamab mafodotin in DREAMM-2 [13, 14, 25] and with other MMAF-containing ADCs [16–19]. In this study, all of the ocular events assessed by both CTCAE and protocol-defined scales, including visual acuity, were managed with dose delays or dose reductions and all resolved within the study period (CTCAE) and/or by last follow up visit (protocol-defined scale). The profile of ocular events including incidence rate and severity were consistent with data from DREAMM-2 [13, 14].

Ocular events were often reported on ophthalmic examination in the absence of subjective symptoms in this study, which is consistent with reports in the DREAMM-2 study [13], and supports the current rationale of ophthalmic examination during treatment to detect ocular events at early stages, if present. Since ocular events are managed frequently with dose reductions and/or delays of belantamab mafodotin, close collaboration with ophthalmologists is beneficial.

Thrombocytopenic events were reported in almost all patients (100%, 4/4 in 2.5 mg/kg cohort; 75%, 3/4 in 3.4 mg/kg cohort) and all events were Grade 3 or Grade 4. These events were manageable through dose reduction and/or dose delay and medical treatment such as transfusion. There were no bleeding events associated with thrombocytopenic events. The proportion of patients in this study who experienced thrombocytopenic events as well as the severity of these events appear marginally higher compared to the DREAMM-2 study [13]. However, the limitation of the small study size must be taken into consideration.

IRRs were observed in 50% of patients in this study (2/4 in 2.5 mg/kg cohort and 2/4 in 3.4 mg/kg cohort). All IRRs were Grade 2 (which compares well with those observed in the DREAMM-2 study [13]), had confirmed recovery within three days, and were managed by prophylaxis and/or post-IRR medication.

Although the number of patients in this Phase I study was limited, the ORRs (50% in 2.5 mg/kg cohort; 25% in 3.4 mg/kg cohort) were consistent with those observed in DREAMM-2 (32%, 2.5 mg/kg) [14, 19]. Clinical responses were generally deep, with all responders (2 of 2) in the 2.5 mg/kg cohort achieving very good partial response (VGPR). Although only 1 patient achieved a PR in the 3.4 mg/kg cohort, there was still a clinical benefit rate of 75%.

Extramedullary disease is often itself refractory to treatment. Notably, of the 3 patients (2.5 mg/kg cohort) in this study with extramedullary disease, 2 achieved VGPR. Though this result is preliminary, this may suggest a possibility of activity of belantamab mafodotin in those patients with unmet clinical need.

Genetic and environmental factors can cause ethnic differences in PK to a varying extent. These ethnic differences may affect dosage, dosage regimen, safety, and efficacy, and can cause a reluctance to rely on clinical data from other ethnic groups for drug approval [21]. Although the PK profiles in Japanese patients in our study were variable, the exposures did overlap with previous results in Western populations. However, the number of Japanese patients was too small to allow for a statistically robust comparison between PK results from Japanese and Western populations [24].

In conclusion, treatment with belantamab mafodotin was tolerated in Japanese patients with RRMM, including those considered to be heavily pre-treated, and demonstrated a manageable safety profile and evidence of clinical activity at both 2.5 and 3.4 mg/kg doses investigated. In comparison to Western patients, no major differences in PK results or the safety profile of belantamab mafodotin were observed. As mitigation strategies for ocular events in close collaboration with ophthalmologists are important for the continuation of patients on belantamab mafodotin based regimens, various combination therapies with varying belantamab mafodotin administration schedules are under development. Additional studies (e.g., DREAMM-7 [NCT04246047] & DREAMM-8 [NCT04484623]) are ongoing to evaluate belantamab mafodotin in combination with well-established and novel agents in patients with RRMM.

### Supplementary Information

Below is the link to the electronic supplementary material.

**Supplementary file. Table S1**: Full inclusion criteria. **Table S2**: Full exclusion criteria. **Table S3**: Ocular event severity grading and mitigation strategy. **Table S4**: Dose reductions (all treated population). **Table S5**: Duration of dose delay (all treated population). **Table S6**: Adverse events of special interest (all treated population). (DOCX 44 KB)

## Data Availability

GSK makes available anonymized individual participant data and associated documents from interventional clinical studies which evaluate medicines, upon approval of proposals submitted to https://www.gsk-studyregister.com/en/. To access data for other types of GSK-sponsored research, for study documents without patient/participant-level data, and for clinical studies not listed, please submit an enquiry via the website.
