# Supplementary material for: Safety, pharmacokinetics, and efficacy of belantamab mafodotin monotherapy in Japanese patients with relapsed or refractory multiple myeloma: DREAMM-11
Source: Int J Hematol. 2023 Sep 5;118(5):596–608. doi: 10.1007/s12185-023-03652-5 (PMC10615937; doi:10.1007/s12185-023-03652-5)
Supplement: Supplementary file 1 — Supplementary file. Table S1: Full inclusion criteria. Table S2: Full exclusion criteria. Table S3: Ocular event severity grading and mitigation strategy. Table S4: Dose reductions (all treated population). Table S5: Duration of dose delay (all treated population). Table S6: Adverse events of special interest (all treated population). (DOCX 44 KB) [file 12185_2023_3652_MOESM1_ESM.docx]

**Safety and Efficacy of Belantamab Mafodotin Monotherapy in Japanese Patients with Relapsed or Refractory Multiple Myeloma: DREAMM-11**

Shinsuke Iida^1^, Kazutaka Sunami^2^, Yuko Mishima^3^, Taku Fujii^4^, Hitomi Kato^4^, Takumi Terao^4^, Yuki Matsuzawa^4^, Mari Matsubara^4^, Timothy Crossman^5^, Brandon E Kremer^6^, Ira Gupta^7^

^1^Department of Hematology and Oncology, Nagoya City University Institute of Medical and Pharmaceutical Sciences, Nagoya, Japan; ^2^Department of Hematology, National Hospital Organization Okayama Medical Center, Okayama, Japan; ^3^Department of Hematology Oncology, Cancer Institute Hospital, Japanese Foundation for Cancer Research, Tokyo, Japan; ^4^GSK, Tokyo, Japan; ^5^Oncology Global Clinical Development, GSK, Stevenage, UK; ^6^Clinical Development, GSK, Upper Providence, PA, USA; ^7^Clinical Oncology, GSK, Upper Providence, PA, USA

# Supporting information

**Table S1.** Full inclusion criteria.

**Table S2.** Full exclusion criteria.

**Table S3.** Ocular event severity grading and mitigation strategy.

**Table S4.** Dose reductions (All treated population).

**Table S5.** Duration of dose delay (All treated population).

**Table S6.** Adverse events of special interest (All treated population).

## Table S1. Full inclusion criteria.

| Inclusion Criteria |
| --- |
| Provided signed written informed consent, which included compliance with the requirements and restrictions listed in the consent form. |
| Male or female, 20 years or older (at the time consent was obtained). |
| ECOG performance status of 0 to 2. |
| Histologically or cytologically confirmed diagnosis of MM as defined according to IMWG 2014 criteria in a patient who fulfilled all of the following: (i) had undergone stem-cell transplant or was considered transplant ineligible; (ii) had received at least 2 prior lines of anti-myeloma drugs containing at least 1 proteasome inhibitor and at least 1 immunomodulator; and (iii) had demonstrated progression on, or within 60 days of completion of the last therapy. |
| Had measurable disease with at least 1 of the following: (a) serum M protein ≥0.5 g/dL (≥5 g/L); (b) urine M protein ≥200 mg/24 hours; and (c) serum FLC assay: Involved FLC level ≥10 mg/dL (≥100 mg/L) and an abnormal serum FLC ratio (<0.26 or >1.65). |
| Patients with a history of ASCT were eligible for study participation provided the following eligibility criteria were met: (a) transplant was >100 days prior to study enrollment and (b) no active infection. |
| Female patients: contraceptive use by women had to be consistent with local regulations regarding the methods of contraception for those participating in clinical studies. A female patient was eligible to participate if she was not pregnant or breast feeding, and at least 1 of the following conditions applied: was not a WOCBP or was a WOCBP and using a contraceptive method that was highly effective (with a failure rate of <1% per year), preferably with low user dependency, during the treatment period and for 4 months after the last dose of belantamab mafodotin and agreed not to donate eggs (ova, oocytes) for the purpose of reproduction during this period. The investigator had to evaluate the effectiveness of the contraceptive method in relationship to the first dose of study treatment. A WOCBP needed to have a negative highly sensitive serum pregnancy test (as required by local regulations) within 72 hours before the first dose of study treatment and agree to use effective contraception during the study and for 4 months after the last dose of belantamab mafodotin. |
| Male patients: Contraceptive use by men had to be consistent with local regulations regarding the methods of contraception for those participating in clinical studies. Male patients were eligible to participate if they agreed to the following from the time of first dose of study treatment until 6 months after the last dose of belantamab mafodotin to allow for clearance of any altered sperm: to refrain from donating sperm and either to be abstinent from heterosexual intercourse as their preferred and usual lifestyle (abstinent on a long term and persistent basis) and agreed to remain abstinent or agreed to use a male condom, even if they had undergone a successful vasectomy, and female partner to use an additional highly effective contraceptive method with a failure rate of <1% per year as when having sexual intercourse with a WOCBP (including pregnant females). |
| All prior treatment related toxicities (defined by NCI-CTCAE, version 4.03), had to be Grade ≤1 at the time of enrollment except for alopecia. Patients with Grade 2 peripheral neuropathy could be enrolled. |
| Adequate organ system function as defined by hematologic (ANC, ≥1.0x10^9^ /L; hemoglobin, ≥8.0 g/dL; platelets, ≥50x10^9^ /L), hepatic (total bilirubin, ≤1.5×ULN [isolated bilirubin ≥1.5×ULN was acceptable if bilirubin was fractionated and direct bilirubin <35%]), renal (eGFR, ≥30 mL/min/1.73m^2^; spot urine [albumin/creatinine ratios via spot urine], <500 mg/g [56 mg/mmol]), and cardiac (LVEF by ECHO, ≥45%; eGFR via MDRD) measures. |
| Abbreviations: ANC, absolute neutrophil count; ASCT, any stem-cell transplant; ECHO, echocardiogram; ECOG, Eastern Cooperative Oncology Group. eGFR, estimated glomerular filtration rate; FLC, free light chain; IMWG, International Myeloma Working Group; LVEF, left ventricular ejection fraction; MDRD, Modified Diet in Renal Disease; MM, multiple myeloma; NCI-CTCAE, National Cancer Institute-Common Terminology Criteria for Adverse Events; WOCBP, woman of childbearing potential; ULN, upper limit of normal. |

## Table S2. Full exclusion criteria.

| Exclusion Criteria |
| --- |
| Systemic anti-tumor therapy within 14 days, or plasmapheresis within 7 days prior to the first dose of study treatment. |
| Symptomatic amyloidosis, active ‘polyneuropathy, organomegaly, endocrinopathy, myeloma protein, and skin changes’ (POEMS) syndrome, active plasma cell leukemia at the time of Screening. |
| Use of an investigational drug within 14 days or 5 half-lives, whichever was shorter, preceding the first dose of study treatment. Prior treatment with a mAb within 30 days of receiving the first dose of study treatment. Prior BCMA targeted therapy. |
| History of an allogeneic stem-cell transplant. |
| Current use of prohibited medications/device or planned use of any of these during the study period. |
| Current corneal epithelial disease except mild punctate keratopathy. |
| Presence of active renal condition (infection, requirement for dialysis or any other condition that could affect patient’s safety). Patients with isolated proteinuria resulting from MM were eligible. |
| Evidence of active mucosal or internal bleeding. |
| Any major surgery within the last 4 weeks. |
| Any serious and/or unstable preexisting medical, psychiatric disorder, or other conditions (including laboratory abnormalities) that could interfere with patient’s safety, obtaining informed consent or compliance to the study procedures. |
| Active infection requiring treatment (antibiotic, anti-viral, or anti-fungal treatment). |
| Evidence of severe or uncontrolled systemic diseases. |
| Malignancies other than disease under study were excluded, except for any other malignancy from which the patient had been disease free for more than 2 years and, in the opinion of the investigators and medical monitor, did not affect the evaluation of the effects of this clinical study treatment on the currently targeted malignancy (MM). |
| Evidence of cardiovascular risk including any of the following: (a) QTcF interval ≥470 msec (the QT interval values had to be corrected for HR by Fridericia’s formula); (b) evidence of current clinically significant uncontrolled arrhythmias, including clinically significant ECG abnormalities such as 2nd degree (Type II) or 3rd degree AV block; (c) history of myocardial infarction, acute coronary syndromes (including unstable angina), coronary angioplasty, or stenting or bypass grafting within 6 months of Screening; (d) class III or IV heart failure as defined by the New York Heart Association functional classification system; and (d) uncontrolled hypertension. |
| Pregnant or lactating female or female who were interrupting lactation. Known immediate or delayed hypersensitivity reaction or idiosyncrasy to drugs chemically related to belantamab mafodotin or any of the components of the study treatment. |
| Known HIV infection. |
| Patients with Hepatitis B were excluded unless the following criteria could be met: HBsAb+ and/or HBcAb+ AND HBsAg- following serology; HBV DNA undetectable at Screening; and monitoring per protocol as well as anti-viral treatment instituted if HBV DNA became detectable. Patients with positive HBsAb alone due to hepatitis B vaccination could be enrolled. |
| Positive HCV antibody test result or positive HCV RNA test result at Screening or within 3 months prior to first dose of study treatment. Patients with positive HCV antibody due to prior resolved disease could only be enrolled, if a confirmatory negative HCV RNA test was obtained. Hepatitis RNA testing was optional and patients with negative HCV antibody test were not required to also undergo HCV RNA testing. |
| Current unstable liver or biliary disease per investigator assessment defined by the presence of ascites, encephalopathy, coagulopathy, hypoalbuminemia, esophageal or gastric varices, persistent jaundice, or cirrhosis. Stable chronic liver disease (including Gilbert’s syndrome or asymptomatic gallstones) or hepatobiliary involvement of malignancy was acceptable if patient otherwise met entry criteria. |
| Previously diagnosed with interstitial lung disease or current complication of interstitial lung disease. |
| Abbreviations: AV, atrioventricular; ECG, electrocardiogram; HBcAb, hepatitis B core antibody; HBsAg, hepatitis B surface antigen; HBV, hepatitis B virus; HCV, hepatitis C virus; HIV, human immunodeficiency virus; HR, heart rate; QTcF, corrected QT interval Fridericia; MM, multiple myeloma; RNA, ribonucleic acid. |

## Table S3. Ocular event severity grading* and mitigation strategy.

| **GRADING** | | | | |
| --- | --- | --- | --- | --- |
| **Measure** | **Grade 1** | **Grade 2** | **Grade 3** | **Grade 4** |
| **Ophthalmic exam findings** | Mild superficial keratopathy (change from baseline) | Moderate punctate keratopathy  and/or  Mild/patchy microcysts  and/or  Mild/patchy Epithelial or stromal edema  and/or  Sub-epithelial haze (peripheral)  and/or  Active stromal opacity (peripheral) | Severe punctate keratopathy  and/or  Diffuse microcysts  and/or  Diffuse Epithelial or stromal edema  and/or  Sub-epithelial haze (central)  and/or  Active stromal opacity (central) | Corneal ulcer |
| **Visual Acuity**^†^ | Change of 1 line from baseline^‡^ | Change of 1 line from baseline^‡^ | Change of 1 line from baseline^‡^ | Change of 1 line from baseline^‡^ |
| **PROPHYLACTIC MEASURES** | | | | |
| **Measure** | **Dose and administration** | | **Timing** | |
| **Preservative-free artificial tears** | Administer in each eye at least 4 to 8 times four times daily. | | Administer daily beginning on Cycle 1 Day 1 until EOT. | |
| **Cooling eye mask** | May apply cooling eye mask to both eyes for approximately 1 hour or as much as tolerated. | | During belantamab mafodotin infusion administration in the first hour for up to 4 hours, as tolerated. | |
| *Grading is based on most severe finding. If eyes differed in severity, protocol-defined scale grading was based on the more severe eye. ^†^Change in visual acuity was due to ocular findings associated with belantamab mafodotin. If change in vision was for reason other than ocular findings, ophthalmic exam findings drove event grading. ^‡^The change in visual acuity by Snellen chart is shown in this table. | | | | |

## Table S4. Dose reductions (All treated population).

|  | **2.5 mg/kg (n=4)** | **3.4 mg/kg (n=4)** | **Total (N=8)** |
| --- | --- | --- | --- |
| **Subjects with Any Dose Reduction,**  **n (%)** | 2 (50) | 3 (75) | 5 (63) |
| **Total Number of Dose Reductions** | 2 | 5 | 7 |
| **Number of Dose Reductions, n (%)** | | | |
| 0 | 2 (50) | 0 | 2 (25) |
| 1 | 2 (50) | 1 (25) | 3 (38) |
| 2 | 0 | 2 (50) | 2 (25) |
| ≥3 | 0 | 0 | 0 |
| Not Evaluable^*^ | 0 | 1 (25) | 1 (13) |
| **Reasons for Reduction, n (%)^†^** |  |  |  |
| AE^‡^ | 2 (100) | 5 (100) | 7 (100) |
| Other | 0 | 0 | 0 |
| **Number of Subjects with Dose Reductions by Dose, n (%)** | | | |
| 1^st^ Dose | 0/4 | 0/4 | 0/8 |
| 2^nd^ Dose | 1/4 (25) | 2/3 (67) | 3/7 (43) |
| 3^rd^ Dose | 0/2 | 2/3 (67) | 2/5 (40) |
| 4^th^ Dose | 1/2 (50) | 1/2 (50) | 2/4 (50) |
| 5^th^ Dose | 0/1 | 0/2 | 0/3 |
| 6^th^ Dose | 0/1 | 0/1 | 0/2 |
| 7^th^ Dose | 0/1 | 0/1 | 0/2 |
| 8^th^ Dose | 0/1 | 0/1 | 0/2 |
| 9^th^ Dose | 0/1 | 0/1 | 0/2 |
| *Not Evaluable was defined for subjects who had C1D1 dose exposure data without subsequent cycles data. ^†^Subjects could be counted multiple times in the same 'reason' row if the subject had multiple reductions for the same reason. ^‡^AE included ocular exam findings as reported as ocular events (protocol-defined scale) as a reason for dose reductions. Per protocol, subjects in the 3.4 mg/mg cohort were allowed a maximum of 2 dose reductions, and subjects in the 2.5 mg/kg cohort were allowed only 1 dose reduction. | | | |

## Table S5. Duration of dose delay (All treated population).

|  | **2.5 mg/kg (n=4)** | **3.4 mg/kg (n=4)** | **Total (N=8)** |
| --- | --- | --- | --- |
| **Subjects with Any Dose Delay, n (%)** | 2 (50) | 3 (75) | 5 (63) |
| **Total Number of Dose Delays** | 3 | 9 | 12 |
| **Time to First Delay (Days), n (%)** |  |  |  |
| 1-21 | 0 | 0 | 0 |
| 22-42 | 0 | 0 | 0 |
| 43-63 | 0 | 0 | 0 |
| >63 | 2 (100) | 3 (100) | 5 (100) |
| Mean (SD) | 292.0 (301.23) | 106.3 (20.50) | 180.6 (182.31) |
| Median (range) | 292.0 (79—505) | 106.0 (86—127) | 106.0 (79—505) |
| **Number of Dose Delays** | | | |
| 0 | 2 (50) | 0 | 2 (25) |
| 1 | 1 (25) | 1 (25) | 2 (25) |
| 2 | 1 (25) | 1 (25) | 2 (25) |
| ≥3 | 0 | 1 (25) | 1 (13) |
| Not Evaluable | 0 | 1 (25) | 1 (13) |
| **Duration of Delays^*^ (days), n (%)** | | | |
| 1-21 | 1 (33) | 0 | 1 (8) |
| 22-42 | 0 | 1 (11) | 1 (8) |
| >42 | 2 (67) | 8 (89) | 10 (83) |
| **Reasons for Delay^†^, n (%)** | | | |
| AE^‡^ | 3 (100) | 9 (100) | 12 (100) |
| Scheduling Conflict | 0 | 0 | 0 |
| Other | 0 | 0 | 0 |
| **Number of Subjects with Dose Delays by Dose , n (%)** | | | |
| 1^st^ Dose | 0/4 | 0/4 | 0/8 |
| 2^nd^ Dose | 1/4 (25) | 1/3 (33) | 2/7 (29) |
| 3^rd^ Dose | 0/2 | 2/3 (67) | 2/5 (40) |
| 4^th^ Dose | 0/2 | 1/2 (50) | 1/4 (25) |
| 5^th^ Dose | 0/1 | 1/2 (50) | 1/3 (33) |
| 6^th^ Dose | 0/1 | 1/1 (100) | 1/2 (50) |
| 7^th^ Dose | 0/1 | 1/1 (100) | 1/2 (50) |
| 8^th^ Dose | 0/1 | 1/1 (100) | 1/2 (50) |
| 9^th^ Dose | 0/1 | 1/1 (100) | 1/2 (50) |
| 10^th^ Dose | 0/1 | 0 | 0/1 |
| 11^th^ Dose | 0/1 | 0 | 0/1 |
| 12^th^ Dose | 1/1 (100) | 0 | 1/1 (100) |
| 13^th^ Dose | 0/1 | 0 | 0/1 |
| 14^th^ Dose | 0/1 | 0 | 0/1 |
| 15^th^ Dose | 1/1 (100) | 0 | 1/1 (100) |
| 16^th^ Dose | 0/1 | 0 | 0/1 |
| 17^th^ Dose | 0/1 | 0 | 0/1 |
| *Duration of delay=actual start date of current dose - expected start date of dose. Expected start date of dose=actual start date of previous dose + 21. ^†^Subjects may be counted multiple times in the same reason row if the subject had multiple dose delays for the same reason. ^‡^AE included ocular exam findings as reported as ocular events (protocol-defined scale) as a reason for dose delays. | | | |

## Table S6. Adverse events of special interest (All treated population).

| **AESIs, n (%)** | **2.5 mg/kg (N=4)** | **3.4 mg/kg (N=4)** | **Total (N=8)** |
| --- | --- | --- | --- |
| **Ocular events (CTCAE)** |  |  |  |
| Any grade | 1 (25) | 1 (25) | 2 (25) |
| Grade 3 | 0 | 0 | 0 |
| Grade 4 | 0 | 0 | 0 |
| **Ocular events (protocol-defined scale)** |  |  |  |
| Any grade | 4 (100) | 3 (75) | 7 (88) |
| Grade 3 | 3 (75) | 3 (75) | 6 (75) |
| Grade 4 | 0 | 0 | 0 |
| **Thrombocytopenia*** |  |  |  |
| Any grade | 4 (100) | 3 (75) | 7 (88) |
| Grade 3 | 4 (100) | 1 (25) | 5 (63) |
| Grade 4 | 0 | 2 (50) | 2 (25) |
| **IRRs** |  |  |  |
| Any grade | 2 (50) | 2 (50) | 4 (50) |
| Grade 3 | 0 | 0 | 0 |
| Grade 4 | 0 | 0 | 0 |
| AESIs, adverse events of special interest; CTCAE, Criteria for Adverse Events; IRRs, infusion related reactions. *Thrombocytopenia includes AEs of thrombocytopenia and platelet count decreased. | | | |
